# Health care providers’ opinions on abortion: a study for the implementation of the legal abortion public policy in the Province of Santa Fe, Argentina

**DOI:** 10.1186/1742-4755-11-72

**Published:** 2014-09-24

**Authors:** Silvina Ramos, Mariana Romero, Agustina Ramón Michel

**Affiliations:** Center for the Study of State and Society (CEDES), Sánchez de Bustamante 27, 1173 Buenos Aires, Argentina; Center for the Study of State and Society (CEDES) and CONICET (National Scientific and Technical Research Council), Sánchez de Bustamante 27, 1173 Buenos Aires, Argentina

**Keywords:** Abortion, Operations research, Opinions, Health care providers, Argentina

## Abstract

**Background:**

In Argentina, abortion has been decriminalized under certain circumstances since the enactment of the Penal Code in 1922. Nevertheless, access to abortion under this regulatory framework has been extremely limited in spite of some recent changes. This article reports the findings of the first phase of an operations research study conducted in the Province of Santa Fe, Argentina, regarding the implementation of the local legal and safe abortion access policy.

**Methods:**

The project combined research and training to generate a virtuous circle of knowledge production, decision-making, and the fostering of an informed healthcare policy. The project used a pre-post design of three phases: baseline, intervention, and evaluation. It was conducted in two public hospitals. An anonymous self-administered questionnaire (n = 157) and semi-structured interviews (n = 27) were applied to gather information about tacit knowledge about the regulatory framework; personal opinions regarding abortion and its decriminalization; opinions on the requirements needed to carry out legal abortions; and service’s responses to women in need of an abortion.

**Results:**

Firstly, a fairly high percentage of health care providers lack accurate information on current legal framework. This deficit goes side by side with a restrictive understanding of both health and rape indications. Secondly, while a great majority of health care providers support abortion under the circumstances consider in the Penal Code, most of them are reluctant towards unrestricted access to abortion. Thirdly, health care providers’ willingness to perform abortions is noticeably low given that only half of them are ready to perform an abortion when a woman’s life is at risk. Willingness is even lower for each of the other current legal indications.

**Conclusions:**

Findings suggest that there are important challenges for the implementation of a legal abortion policy. Results of the study call for specific strategies targeting health care providers in order to better inform about current legal abortion regulations and to sensitize them about abortion social determinants. The interpretation of the current legal framework needs to be broadened in order to reflect a comprehensive view of the health indication, and stereotypes regarding women’s sexuality and abortion decisions need to be dismantled.

**Electronic supplementary material:**

The online version of this article (doi:10.1186/1742-4755-11-72) contains supplementary material, which is available to authorized users.

## Background

In Argentina, abortion has been decriminalized under certain circumstances since the enactment of the Penal Code in 1922. Nevertheless, almost a century after this legal piece went into effect, access to termination of pregnancy under this regulatory framework is extremely limited.

An estimated 400,000 clandestine abortions take place in Argentina each year, more than one abortion for every two live births[1, 2]. While these figures suggest the ineffectiveness of criminalization as a strategy to discourage women from terminating their pregnancies, information on maternal morbidity and mortality clearly show the adverse consequences that these abortions – most of which are unsafe as a consequence of illegality – have for women’s health and lives. Official statistics indicate that more than 50,000 women are discharged from public hospitals each year for complications stemming from unsafe abortions^a^, and for two decades abortion complications have been the third cause of discharge due to causes related to pregnancy, delivery, and puerperium at the national level[3].Abortion has been the leading cause of maternal mortality since 1980. Moreover, in the 2008–2012 quinquennium it represented 20% of all maternal deaths[3], much higher than the 12% estimated for the Latin American and Caribbean region[4]. The maternal mortality rate (MMR) reported by the National Ministry of Health of Argentina for 2012 was 35/100,000 live births[5]. To continue the descending rate observed from 1990 until present, the MMR for 2015 is estimated at 40/100,000 live births. This trend indicates that the rate will be 2.3 times higher than the commitment of the country with Millennium Development Goal 5 (MDG 5)[6].

Like most Latin American and Caribbean countries, Argentina has a moderate restrictive legal framework concerning the voluntary termination of pregnancy. The Penal Code establishes abortion as an offense against life, and sets specific circumstances under which the practice is permitted (referred to in the country as “legal abortions”). Article 86 of the Code rules that “An abortion performed by a licensed physician with the consent of the pregnant woman is not punishable: 1^st^ If it has been done to avoid danger to the life or health of the mother and if this danger cannot be avoided by other means. 2^nd^ If the pregnancy is the product of a rape or indecent assault against an idiot or demented woman. In this case, her legal representative’s consent shall be required for the abortion.” This statutory allows abortion in three exceptions: life risk, health risk, and when the pregnancy results from rape. The latter includes a generic permission as well as a specific one for women with mental disabilities. The lack of access to legal abortions has been the rule for more than nine decades. In the last years this scenario has started to change[7]. One of the key factors that have hindered access to non-punishable abortion is the contentious and restrictive interpretation of the legal text. Both the scope of the health indication as well as whether the rape indication refers only to women with mental disabilities or whether it refers to any woman who has been raped have been the core of the dispute. The restrictive view of legal abortion has been reproduced in the training of health professionals[8]. Another factor that accounts for the narrow interpretation is the fact that abortion is still socially perceived as an offense, making the exceptions of Article 86 almost invisible. Finally, the lack of enforcement of a health policy has contributed to the barriers to access legal abortions as well[8].

In Argentina the information regarding legal abortions performed by the healthcare system is scant and extremely scattered. According to information from the media and personal communications with healthcare services, very few women who qualify for a legal abortion receive information about this option and/or receive care[9].

The lack of an effective healthcare policy at both the national and provincial levels has become evident by cases that were made public in recent years[9]. Since 2001, the media has echoed cases of women with anencephalic fetuses, girls and adolescents with pregnancies resulting from rape, and women with serious physical health problems who received evasive responses from healthcare authorities, healthcare services, and health care providers[9]. At the same time, these women were victims of threatening actions by “anti-choice” groups, and endured unnecessary judicial instances to obtain authorization and access abortion[9].

In response to these cases with strong repercussions in public opinion, the National Program on Sexual Health and Responsible Procreation of the Ministry of Health, requested a team of specialists to draft a Guide for the Comprehensive Care of Legal abortion, released in November 2007^b^.

However, the situation is very different in each province: lack of regulation for the provision of legal abortions; protocols that impose unnecessary barrier-generating requirements; multiple challenges for implementing services; political volatility coming from the fragility of Government commitment towards the issue[7].

### Operations research to inform healthcare policy

Given the changes that have taken place in Argentina in recent years, it is a strategic and timely moment to strengthen the political and institutional conditions required for the design and implementation of a legal abortion policy. Furthermore, the effective implementation of these policies – along with other healthcare interventions – is essential to bringing Argentina closer to achieving the MDGs that involve maternal mortality reduction and universal access to reproductive healthcare services.

This article reports the findings of the first phase of an operations research study in healthcare services[10–12]. Its objectives are a) to generate information regarding the institutional context in which public policy takes place; b) to strengthen both the institutional capacity and technical ability of health teams, and c) to foster their abilities to act as agents of change, creating friendly and high quality services for women in need of a legal abortion[13].

In order to accomplish these objectives, the project combines research and training to generate a virtuous circle of knowledge production, decision-making, and the promotion of informed healthcare policy[14].

Research into healthcare systems and policies has shown that knowledge of local contexts and actors’ views are crucial to guide interventions in services aimed at modifying the *status quo*[15, 16]. It has also been shown that the involvement of actors in the process of change improves the opportunities for effective and sustainable policy implementation[17–19].

## Methods

This is an operations research using a pre-post design, organized into three phases: baseline, intervention, and evaluation of results[20].

The research protocol was approved by the Scientific and Ethical Review Group (SERG) of the RHR-WHO, a national ethics committee^c^, and a committee dependent upon the healthcare authorities of the municipality.

The study was conducted in two public hospitals in an Argentine province^d^. The initial stage elaborated a baseline of health teams’ abortion-related knowledge, attitudes, and practices. This baseline was key to adapt a curriculum to sensitize and create enabling conditions to comply with current legal context and women’s needs. It also provided information to evaluate the results of our intervention over time^e^.

The two hospitals, part of the province’s public health network, are third level referral general hospitals. Both are teaching facilities with Ob-Gyn departments. They have an average of 1,800 deliveries and 200 admissions for incomplete abortions annually. They were selected based on the fact that, at the time of project approval, they were beginning an internal process aimed at implementing a policy to deal with legal abortion care. Meetings were held with representatives of the Provincial Ministry of Health, hospital authorities and heads of healthcare services, as well as with health teams. The goal of these meetings was to frame the project, discuss its scope, obtain the approval of the healthcare team^f^, and create receptive conditions for fieldwork.

The baseline applied qualitative and quantitative techniques. An anonymous self-administered survey was distributed to all of the staff (physicians and non-physicians) from the Ob-Gyn services in both hospitals. The questionnaire, which had 25 questions – 24 of them with multiple choice answers and 1 open – collected information about the socio-demographic characteristics of the respondent, opinions towards decriminalization of abortion, knowledge about abortion’s legal situation in the country, perceptions about the necessary conditions to perform legal abortions and their professional experience, and willingness to perform legal abortions. The survey, informed consent, and an envelope were given to the professionals by a staff member of each hospital. Each hospital had a sealed box to return the survey in the envelope and the informed consent separately. The boxes remained in the services during December 2010. All the Ob-Gyn staff in the two hospitals was invited to participate, a total of 240 people. 157 surveys were obtained, which corresponds to a response rate of 65.4%.

The semi-structured interview was applied to professionals with a strategic role in the decision-making process in the Ob-Gyn services, and other services relevant to abortion care – anesthesiology, mental health and clinical training. The interview looked into the personal and services’ experiences with abortion, knowledge and opinion of the legal framework, willingness to perform abortions permitted by law, and the services’ regulations for access to these abortions. They were carried out in the interviewees’ workplaces. Informed consent was applied and authorization was obtained to record them. A convenience sample was utilized, comprising 27 members of the two institutions.

The interviews were transcribed. Data reduction was performed manually and responses were put into text tables showing each interviewee’s response to each question. The self-administered questionnaires were edited and added to a database using the program SIPEwin^g^. The survey analysis utilized simple frequencies, double entry tables, and X^2^ and R tests when it was necessary to estimate the statistical significance. The responses were analyzed by sex and profession and only reported when statistically significant differences were observed.

## Results and discussion

### A platform for change: knowledge, attitudes, and practices surrounding abortion

Our interest in analyzing knowledge, attitudes, and practices surrounding abortion in public hospitals has various reasons. First, knowledge about health teams’ perspectives towards abortion is relevant for the implementation of abortion healthcare policy; this evidence is scarce in Argentina. Health care professionals are policy’s gatekeepers who facilitate or hinder access to voluntary termination of pregnancy, thus becoming a key actor in improving abortion related services[21]. Furthermore, the effective implementation of access to legal abortions requires changes at various levels of the system, including organizational culture and providers’ knowledge, attitudes, and practices.

Second, health care providers have professional capital (experience, social and political relationships, technical competence), giving them legitimacy and placing them as an authoritative voice within the healthcare field for the common good of society[22–24]. Finally, because the history of law liberalization processes and implementation of abortion policies shows that this professional community has always played a key role as an interest group[25–30].

The self-administered survey revealed the opinions of 157 members of the staff in both hospitals, 75% of which were women. The average age was 39 years old (ranging from 22 to 69); 4 of every 10 were physicians and 3 of every 10 were nurses. The interviews collected the opinions of 27 health care providers; 17 women and 10 men; 23 physicians, 2 social workers, 1 nurse, and 1 psychologist.

#### Knowledge of the legal framework: uncertainty and confusion

The survey inquired about the knowledge of the legal framework, aimed at observing to what extent the healthcare teams had appropriate information and understanding of current legal norms in Argentina (Table 1).

**Table 1:** **Statements considered as correct or incorrect according to the current law (percentages)**

	Correct	Incorrect	Unsure/no response	Total
The Penal Code permits abortion in case of risk to woman’s life	79.7	8.2	12.0	100
The Penal Code permits abortion in case of rape of a woman with a a mental disability	75.9	13.3	10.8	100
The Penal Code permits abortion in case of risk to woman’s physical health	47.5	31.6	20.9	100
The Penal Code permits abortion in case of risk to woman’s mental health	19.6	56.3	24.1	100
The Penal Code permits abortion for any woman who has been raped	16.5	64.6	19.0	100

Only two out of the four current grounds for legality –danger to the woman’s life and rape of a mentally disabled woman –were identified by the majority of the respondents, showing a precarious knowledge of legal framework. Additionally, the proportion of those who incorrectly mentioned that risk for physical or mental health, or rape of any woman were not included among legal indication, rated up to 5–8 of every 10.

The overwhelmingly restrictive interpretations and weak knowledge regarding current legal norms can be understood in the light of the cultural climate in which health care providers are trained and socialized. In the interviews they reflected on this situation and pointed out determinants such as the failure to address abortion during training, narrow discourses, and the discretional nature of institutional decision-making, inconsistency and uncertainty of the legal framework.

*And now I realize that the topic of legal abortion wasn’t very clear because they hadn’t taught me this topic very well; when I took a course on legal medicine they didn’t teach it very well.*

*Because until now abortion has been a concealed topic… the figures are hidden, there aren’t statistics or monitoring.*

*The legislation is confusing… it’s very confusing, and I think that it’s not complied with because there’s a widespread lack of awareness – myself included – about this legislation. In all these years it’s not a topic that we have dealt with.*

*They always put us in a pickle, because the laws aren’t clear…*

#### Personal stances on the decriminalization of abortion: a few steps ahead of the current legal framework

The personal stances on the decriminalization of abortion were collected in the survey by listing specific circumstances and asking respondents to indicate their level of agreement. Despite the respondents’ limited knowledge, the results show that the personal stances closely mirror the abortion legal status. The majority of respondents backed all of the indications. Nevertheless, the indication for risk to mental health had less support. The two minorities (less than 2 of every 10) were those who agreed with the most permissive exception –the autonomous decision of the woman– and those who agreed with total criminalization.

These results are similar to studies that have been conducted regarding the community at large in Argentina and other countries in the region[24, 31–37]. According to a review of the literature in Latin America, the majority of healthcare professionals does not completely agree or disagree with the exceptions, rather their stances depend on the type of indication and are less favorable to the decriminalization of abortion when the indication would entail an increase in women’s autonomy[38]. In a survey of gynecologists and obstetricians conducted in Mexicoª, 93% agreed that abortion should be legal in case of danger to woman’s life. This percentage decreased somewhat in case of risk to health, reaching 83%, with 82% in agreement in case of severe congenital malformations. A similar proportion responded that abortion should be legal in case of rape[32]. In Peru, a survey showed similar results. The study reported high rates of agreement with regard to risk to life (97%), risk to health (89%), rape (80%) and fetal malformations (86%), but revealed substantially less agreement for socio-economic conditions (20%)[35].

The level of agreement observed in this study is alike to those registered in public opinion surveys in Argentina in recent years. These surveys show a majority level of agreement that is stable and consistent for the life, health and rape indications, whereas agreement with the exception for mental health and/or the woman’s decision is substantially less[31, 39].

While the survey results show a pattern that generally follows the parameters of current legislation, the interviews offer information that contribute to hypothesize obstacles to the provision of legal abortion. On one hand, some professionals’ interpretations regarding the indications for health and rape are influenced by gender stereotypes, even when the personal stance regarding the exception is positive.

*There are going to be lots of people who say that someone who was raped no longer needs a police report, that you don’t need to prove it (…) lots are going to say ‘go to the police and file the report, because they’re going to take care of it’; every law has a loophole […]*

Personal stances are less receptive to mental health indication. This could be explained by the mainstream vision, which tends to pursue a diagnosis and levels of harm, whereas mental health does not;

*How do you demonstrate mental or psychosocial imbalance? It’s very difficult to demonstrate; it’s easier to demonstrate high blood pressure; you take it and it’s done. But there are many things that one can’t know, so the problem is going to be that there are lots of women who are going to say, “fine, go to the hospital and tell them that you’re very anxious about the pregnancy, and they’re going to take care of it”.*

*[…] The thing is that we can all see how women’s health has to do with what’s social, psychological, not just what can happen to the body if the woman goes forward with her pregnancy or not. The majority of us doctors see the situation like that, but when we have to act on certain things… we see it more biologically. So we constrain ourselves to the biology, the body, we don’t see the rest, mental health, social health and so on.*

The opinions in favor of decriminalization on the grounds of women’s request are a minority, and based on different notions. First, they are backed by the idea that women’s lack of responsibility in sexuality and reproduction would bring them to terminate if access is facilitated.

*I think that one has to put a lot into thinking about whether the woman’s life is at risk or not. Because in reality she might seeks an abortion just because she’s trying to get one, without having to jump through too many hoops […] so there will be women who say, “Why am I going to use contraception? If I get pregnant I’ll get an abortion in the hospital and that’s that.” It is a double-edged sword, because there are going to be lots of women who use abortion as a method of contraception.*

Additionally, the weak support in favor of decriminalization of abortion when it is a woman’s decision seems to rest on the idea that the life in gestation is relevant and can be displaced only within certain limits.

*I don’t agree with non-punishment in cases of rape or in cases of an idiot woman; I do in the case of anencephaly, but I don’t agree with ending the life of that child. No matter if it was rape, it’s a person who’s going to live.*

Although some agree with decriminalization when it’s a woman’s decision, personal involvement seems to be limited, certainly imprecise, as the following testimony illustrates:

*I would think that it should be decriminalized. On the other hand I don’t that I would perform an abortion on anyone who comes and asks for one just because. But I think that they should have the right to have one if they can’t take care of their child, or whatever reason … I think that if they decide, women should be listened to and it shouldn’t be criminalized. Anyway I insist, I wouldn’t do one “just because”, but it should be available to whoever seeks one.*

Despite the low agreement observed with abortion on request, some interviewees highlighted the need for a more liberalized regulation. Despite ambiguity, these testimonies suggest the recognition for women to have the right to make decisions about their bodies, as well as to the social inequality implicit in restrictive legislation.

*I think that they have to legalize abortion; I’m totally in agreement with legalizing abortion, which is currently penalized. It’s a woman’s decision. It’s another thing if someone is in disagreement, but it’s a personal question; it doesn’t have to become known throughout the community. Abortion has to be legalized because of this, because it always becomes a question of purchasing power. She has what it takes to do it, does it, and she who doesn’t has to die because of the conditions, because if she’s decided to do it, she’s going to do in the worst conditions, and even if they tell her she’s going to die, she’s going to do it.*

*The truth is that I think it should be even more open that it already is. Abortion should be legal. It’s as though you ask for contraceptive methods and you got pregnant and you don’t want it; you should be able to ask for an abortion in good conditions, health conditions, with all the controls that are required and so that you don’t end up doing whatever. And someone who has money does it in good conditions and those who don’t have money do whatever barbarity. Because the truth is that they’re imbeciles, a patient comes in with a botched abortion, here we finish doing it well and nobody criticizes her; you have to finish a job badly done but not start it. This is crazy […] I think the situation of abortion is horrible for any woman […][But…] in reality the woman who decides to have an abortion is going to do it anyway, whether it’s legal or illegal.*

*Regarding liberalizing the law, guaranteeing that people have good care, regardless of the decision made, the decision is personal […]. I think that every individual has to have the freedom to be able to decide about their life, and that the State guarantees the health of that patient […].*

The agreement with the decriminalization of abortion in different circumstances was also analyzed to evaluate the consistency of the interviewee’s opinions. The responses were re-coded to “in agreement” and “in disagreement” in order to build a typology of personal stances that had the specific combination of each respondent in mind, and in which each category was exclusive with respect to the rest, which permitted a clear identification of each respondent in each category (Table 2).

**Table 2:** **Agreement with the decriminalization of abortion in certain circumstances (percentages)**

	The law should penalize all circumstances (*)	Risk to the woman’s life	Risk to the woman’s physical health	In case of rape	Risk to the woman’s mental health	The woman’s decision
**The law should penalize all circumstances**	100	1,1 (*)	1,4 (*)	1,5 (*)	2,2 (*)	0,0 (*)
**Risk to the woman’s life**	20,0	100	100	92,5	97,8	100
**Risk to the woman’s physical health**	20,0	80,5	100	83,6	97,8	95,7
**In case of rape**	20,0	71,3	80,0	100	84,8	95,7
**Risk to the woman’s mental health**	20,0	51,7	64,3	58,2	100	87,0
**The woman’s decision**	0,0	26,4	31,4	32,8	43,5	100

(*) Not statistically representative. Less than 10 cases.

The results show perspectives that are consistent in those who have more conservative opinions as well as those who have more permissive opinions, the two minorities who are referred to above. In those who revealed conservative perspectives, at least 8 (or more) of every 10 also agree with life and physical health indications. In those who revealed move permissive perspectives, 9 (or more) of every 10 who agree with the liberalization of abortion on the grounds of being a woman’s decision.

At the same time, all of the interviewees had majority agreement with the indications that were most restrictive (risk to health and physical health), and are less in agreement with other exceptions like risk to mental health and a woman’s independent decision. Regardless, it can also be observed that when the support for the exceptions for mental health and rape is higher, support is also higher for a woman’s independent decision.

When these results are analyzed by sex, it becomes clear that women tend to concentrate themselves in the category “risk to life or physical health” (47.4%). Only 24% support the social indication and woman’s independent decision. Within men, in contrast, 50% are included in these two latter indications.

#### Willingness to perform abortion: a half-baked commitment

**Figure 1:**
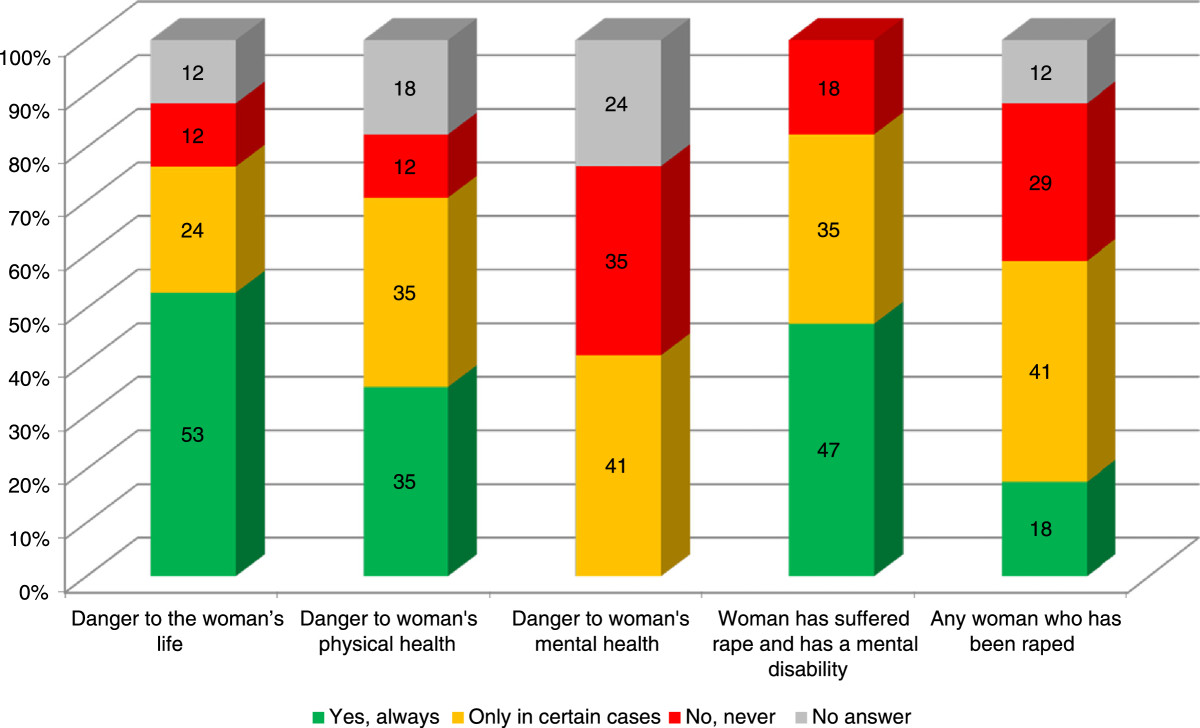
**Willingness to perform a legal abortion by cause (percentages).**

The willingness to terminate a pregnancy was revealed in a question that listed specific circumstances and respondents marked the options always, only in some cases, never, and no response. A double analysis of this information is presented. First, the willingness of healthcare professionals to perform an abortion under legal conditions is considered (Figure 1).

Health care providers’ willingness to carry out abortions permitted by law is not very positive. 5 of every 10 respondents reported that they were willing to perform an abortion in all cases of risk of a woman’s life and rape of mentally disabled woman. Moreover, willingness is noticeably less when physical health is at risk: 3 out of 10 respondents said they would always perform an abortion under these circumstances. 4 of every 10 are willing to perform an abortion when there is a risk to mental health or when a woman was raped, but will not do it in all cases.

#### Requirements for abortion provision in the healthcare service

**Figure 2:**
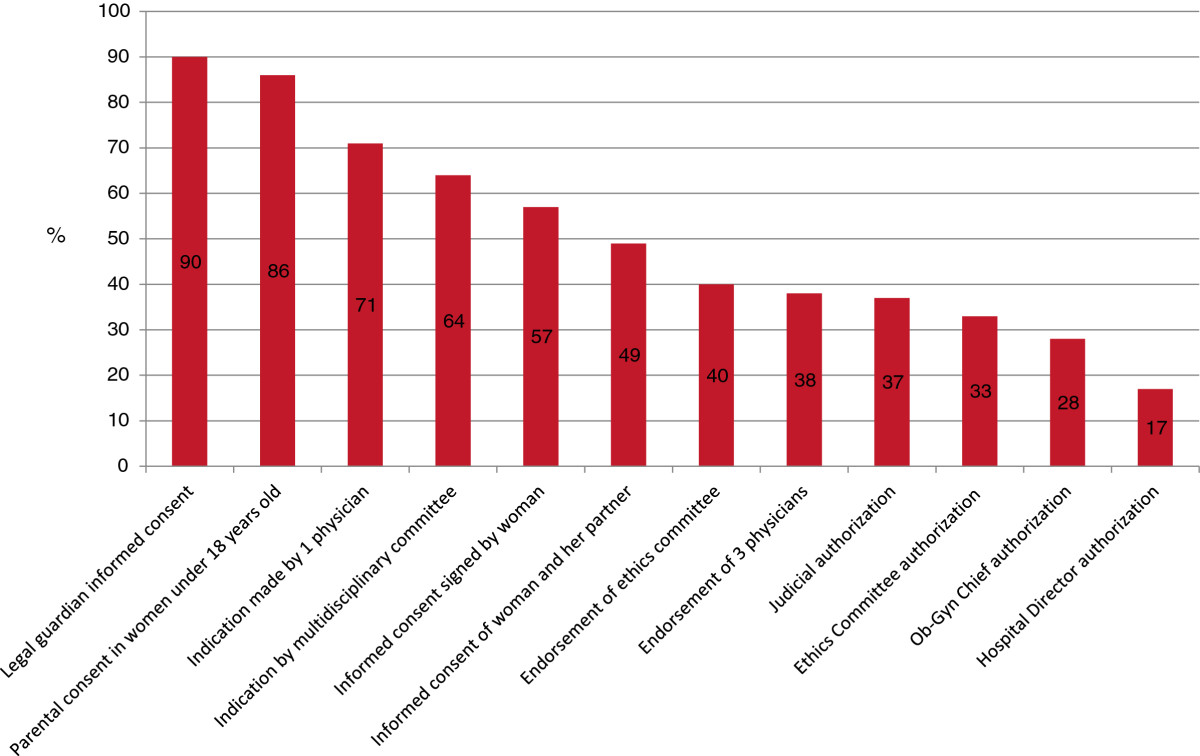
**Report of requirements perceived as necessary to perform a legal abortion (percentage).**

The study also looked into the requirements perceived as necessary by healthcare professionals to perform legal abortions in order to assess feasibility of non-punishable-abortion policies (Figure 2). A series of requirements was listed, including those covered by the current legal framework.

The respondents perceived the most important requirements to be those covered by the current national guide, suggesting a positive environment to implement current policy. In this sense, only 3 out of 10 respondents mentioned judicial or ethics committee authorizations are required. Nevertheless, half of the respondents indicate that informed consent should come from the couple as well as the woman.

#### Barriers to access: fear and ignorance at the frontline

The survey explored respondents’ opinions about barriers to access legal abortions in their services. 7 of every 10 indicated that health care providers do not perform legal abortions because of fear of legal consequences, and not being sufficiently familiar with the procedure; 6 of every 10 answered that healthcare professionals do not perform abortions due to restrictive interpretation of the legal framework, the hospital administration requires judicial authorization, and they are not familiar with the indications. Additionally, 5 of every 10 responded that healthcare professionals do not perform abortions because of judicial interference. Finally 4 of every 10 responded that physicians do not perform abortions permitted by the law because they declared themselves as conscientious objectors.

The interviews reveal that personal stances also act as barriers to access to abortions permitted by law.

*Because this topic generates resistance, it’s simple, for lack of knowledge and personal resistance […]. Society is still not prepared. We’ll prepare ourselves a little at a time if we get ourselves to debate about and reflect on this.*

*[…] I think that all of these things happen because of ignorance, for lack of information, like a doctor asking a judge for authorization to perform an abortion when in reality it’s not needed. So that’s where the bureaucracy is, that ends up… or rather, the result is that the pregnancy continues, and you’re not going to perform an abortion if the pregnancy is far along.*

*I think that doctors put a ton of things between the patient or woman and her health, things that we shouldn’t put there: ideology, culture, religion, personal questions, and the rest. Care has to be equal, without casting judgments, or at least that’s how we’re sworn in; the Hippocratic oath is what I’m referring to, but oh well…*

## Conclusions

This study can be considered a pioneer attempt to inform a specific public policy aimed at improving access to legal abortion in a province of Argentina. Its findings suggest that there are important challenges for the implementation of such a policy. As the literature has shown in other contexts, the role of health care providers is critical for the feasibility of those policies and much of what needs to be reinforced is directly related to the knowledge, attitudes, and practices of such gatekeeper and public opinion actors towards legal abortion access.

This diagnosis revealed that a fairly high percentage of health care providers lack accurate information on current legal framework, and that this deficit goes side by side with a restrictive understanding of both health and rape indications. The former is restricted to physical health, whereas rape is mostly acknowledged only when a woman has mental disability. Secondly, while a great majority of health care providers support abortion under the circumstances that correspond with the Argentine legal framework, most of them are reluctant to accept abortion on request. Thirdly, health care providers’ willingness to perform abortions -even in those circumstances they support- is noticeably low given that only half of them is ready to perform an abortion when a woman’s life is at risk. Willingness is even lower for each of the other current legal indications.

This scenario calls for specific strategies targeting health care providers in order to better inform about current legal abortion regulations and to sensitize them about abortion social determinants. The interpretation of current legal framework needs to be broadened in order to reflect a comprehensive view of the health indications, and stereotypes regarding women’s sexuality and abortion decisions need to be dismantled.

Until very recently, access to legal abortion was extremely restricted in Argentina. The difficulties with access were due to multiple factors, including a deficient knowledge of the legal framework, a narrow understanding of current legal indications, a weak conscience regarding professional responsibility, a lack of running policies, and a social and political debate highly focused on the legal reform. Given this context and barriers such as health care delays, contestations, and undue administrative procedures, not to mention denied abortions, have severely undermined legal abortion-related services.

Since 2006, policy and political scenarios have slowly started to change. Recently, there has been some development of healthcare protocols at both national and provincial levels together with some healthcare services’ willingness to provide abortion related care. Additionally, there have been judicial decisions to guarantee access to legal abortion, including two Supreme Court rulings, which have contributed to a more liberal interpretation of the indications established in the penal code. International human rights bodies have made recommendations to the Argentine government, such as considering that restricting women’s access to an abortion could entail cruel, inhuman, or degrading treatment.

This study is part of a larger dialogue that has been taking place within the community of healthcare providers, stakeholders, policy makers, researchers, women’s groups, human rights activists, lawyers, and politicians in order to advance women’s access to legal and safe abortion. In this context, we consider that this study is a contribution to the reinforcement of legal abortion policy in the Province of Santa Fe since it provides an accurate diagnosis of those who are meant to play a key role in the implementation of that policy.

## Endnotes

^a^The statistics on hospital discharges published by the National Ministry of Health only include institutions within the public sector and do not report on private or social security institutions.

^b^By that time, only three jurisdictions in Argentina had similar regulations: the province of Buenos Aires, the Autonomous City of Buenos Aires, and the city of Rosario. Nevertheless, these regulations included a restrictive interpretation of the rape exception, defining that only a woman with several health disability was entitled to get a legal abortion.

^c^Ethics Research Committee of the Center for Medical Education and Medical Research “Norberto Quirno” (in Spanish, the *“Comité de Ética en Investigación de CEMIC (Centro de Educación Médica e Investigaciones Médicas “Norberto Quirno”*)), recognized by the Office for Human Research Protections, U.S. Department of Health and Human Services (HHS).

^d^To preserve the anonymity of the participants, the participant hospitals are not identified.

^e^Adaptation of the curricula “Exceptions for health. Legal pregnancy termination, ethics, and human rights” (in Spanish, *“Causal salud. Interrupción legal del embarazo, ética y derechos humanos.* La Mesa por la Vida y la Salud de las Mujeres y la Alianza Nacional por el Derecho a Decidir.http://www.clacaidigital.info:8080/xmlui/handle/123456789/152 (accessed September 23 2014).

^f^The entrance permission had already been obtained when the project was being prepared for presentation to the Call for Proposals of HRP/WHO through consultations with authorities from the services and the ministry. Nevertheless, it was deemed necessary to confirm this with the research subjects before initiating the project, involving as well the health care teams of both institutions.

^g^The program SIPE (in English, Interview Processing System) (SIPEwin in its Windows version) was developed in 1986 by Javier Babino and Juan Volkis.

## Authors’ information

Silvina Ramos, sociologist. Senior researcher at CEDES (Center for the Study of State and Society), Buenos Aires, Argentina (http://www.cedes.org).

Mariana Romero, MD MSc. Senior researcher at CEDES (Center for the Study of State and Society) and adjunct researcher at CONICET (National Scientific and Technical Research Council, Buenos Aires, Argentina.

Agustina Ramón Michel, lawyer. Adjunct researcher at CEDES (Center for the Study of State and Society), Buenos Aires, Argentina (http://www.cedes.org).

## Authors’ original submitted files for images

Below are the links to the authors’ original submitted files for images. Authors’ original file for figure 1 Authors’ original file for figure 2
