# Supplementary figures and images for: Health care providers’ opinions on abortion: a study for the implementation of the legal abortion public policy in the Province of Santa Fe, Argentina
Source: Reprod Health. 2014 Sep 24;11:72. doi: 10.1186/1742-4755-11-72 (PMC4197265; doi:10.1186/1742-4755-11-72)

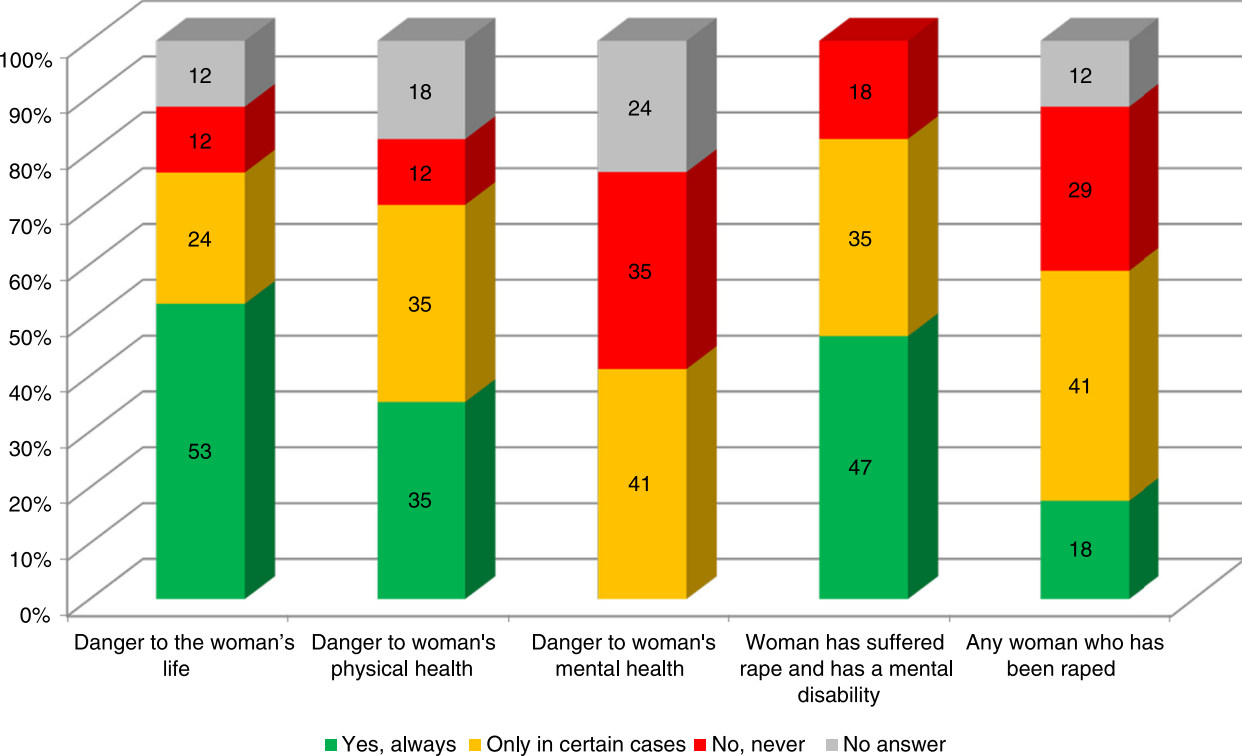

Supplement: Supplementary file 1 — Authors’ original file for figure 1 [file 12978_2014_323_MOESM1_ESM.pdf]

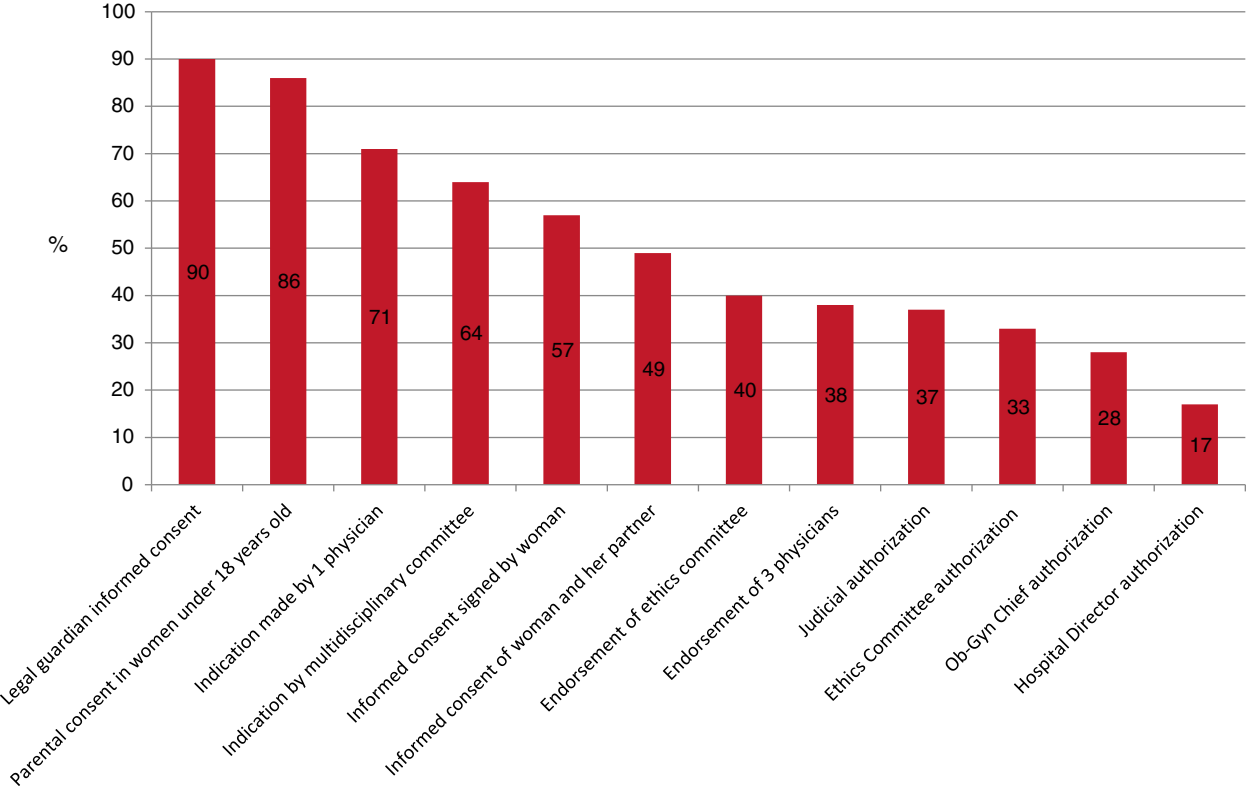

Supplement: Supplementary file 2 — Authors’ original file for figure 2 [file 12978_2014_323_MOESM2_ESM.pdf]
